# CoFe_2_O_4_-Modified Biochar Is Associated with Microbial Community and Antibiotic Resistance Gene Profiles in Vertical Flow Constructed Wetlands Exposed to Oxytetracycline

**DOI:** 10.3390/microorganisms14091950

**Published:** 2026-09-03

**Authors:** Yiping Guo, Meng Bai, Yan Liu, Leicheng Li, Shihang Ni, Camilo Ayra-Pardo, Wei Yuan

**Affiliations:** 1 School of Ecology and Environment, North China University of Water Resources and Electric Power, Zhengzhou 450046, China; 2 CIIMAR/CIMAR LA, Interdisciplinary Centre of Marine and Environmental Research, University of Porto, Terminal de Cruzeiros do Porto de Leixões, Avda. General Norton de Matos s/n, 4450-208 Matosinhos, Portugal; cayrapardo@ciimar.up.pt

**Keywords:** oxytetracycline, antibiotic resistance genes, microbial community structure, CoFe_2_O_4_-modified biochar, vertical flow constructed wetlands

## Abstract

The widespread occurrence of antibiotics in aquatic environments has raised concern over the dissemination of antibiotic resistance genes (ARGs). Although vertical flow constructed wetlands (VFCWs) efficiently remove antibiotics from wastewater, the relationships between substrate configuration, microbial communities, and ARG abundance remain poorly understood. Here, five laboratory-scale VFCWs with different substrate configurations, including cobalt ferrite (CoFe_2_O_4_)-modified biochar and its composite with zeolite, were evaluated using synthetic domestic wastewater amended with oxytetracycline (OTC). CoFe_2_O_4_ modification increased the specific surface area and pore volume of biochar and altered its adsorption behavior. All VFCWs achieved high OTC removal (>97%), despite differences in OTC accumulation within the substrate. In contrast, ARG and mobile genetic element abundances and bacterial community profiles varied among reactors. Combined *tetA* and *tetX* abundance ranged from 2.55 × 10^2^ to 1.90 × 10^5^ copies g^−1^, with the CoFe_2_O_4_-modified biochar–zeolite reactor showing the lowest mean ARG abundance. Exploratory co-occurrence analysis identified associations of *Acinetobacter* with *tetA* and *Flavobacterium* with *tetA* and *tetX* (nominal Spearman *p* < 0.05), although these correlations do not establish ARG host identity or horizontal gene transfer. Overall, the findings indicate that microbial ecological patterns associated with ARG abundance may differ among VFCWs even when antibiotic removal efficiencies are comparable.

## 1. Introduction

Antimicrobial resistance (AMR) has become one of the most pressing global public health challenges, with aquatic environments increasingly recognized as important reservoirs for antibiotic-resistant bacteria (ARB), antibiotic resistance genes (ARGs) and mobile genetic elements (MGEs) [1,2,3,4,5]. The widespread use of antibiotics in human medicine, livestock production and aquaculture has resulted in their continuous release into wastewater, where residual concentrations—even at sub-inhibitory levels—can exert selective pressure on microbial communities and promote the persistence and dissemination of resistance determinants [1,3,6,7,8]. Consequently, wastewater treatment systems are no longer viewed solely as facilities for contaminant removal but also as complex microbial ecosystems that can influence the environmental fate of ARGs and their associated microbial hosts [4,5,8]. Understanding how treatment technologies affect these microbial ecological processes is therefore essential for evaluating their contribution to mitigating antimicrobial resistance.

Constructed wetlands (CWs) have emerged as sustainable and cost-effective wastewater treatment technologies because they integrate physical filtration, adsorption, plant uptake and microbially mediated degradation within a single engineered ecosystem [9,10]. Among them, vertical flow constructed wetlands (VFCWs) offer improved oxygen transfer and have demonstrated high removal efficiencies for nutrients, organic contaminants, and a wide range of antibiotics [10,11,12,13]. Nevertheless, pollutant removal efficiency alone provides only a partial measure of treatment performance. Several studies have reported substantial reductions in antibiotic concentrations accompanied by decreases in ARG abundance, whereas others have observed the persistence—or even enrichment—of ARGs despite efficient contaminant removal [5,13,14,15,16]. These contrasting observations suggest that microbial ecological responses cannot be inferred directly from antibiotic removal efficiency and raise a fundamental question: can wetlands exhibiting comparable antibiotic removal efficiencies nevertheless differ in their microbial ecological performance? Addressing this question requires a better understanding of the factors governing microbial community assembly and ARG abundance within constructed wetlands.

One factor that may contribute to these contrasting microbial ecological outcomes is the wetland substrate [13]. Beyond providing structural support, substrates function as engineered microbial habitats, regulating biofilm development, oxygen availability, nutrient gradients and microbial colonization while simultaneously controlling contaminant adsorption [9,15,16]. Consequently, modifying substrate physicochemical properties may alter microbial community assembly and the abundance of ARGs independently of overall pollutant removal [13,17]. Biochar has received considerable attention as a wetland substrate because of its porous structure, high specific surface area and favorable adsorption characteristics [18,19]. More recently, biochar modification using metal oxides has been explored to enhance these physicochemical properties further. Among these materials, cobalt ferrite (CoFe_2_O_4_) exhibits high chemical stability, magnetic recoverability and abundant surface-active sites, making it a promising modifier for environmental remediation [20,21]. While CoFe_2_O_4_-modified materials have been increasingly investigated as adsorbents for antibiotics and other organic pollutant removal [22,23], their influence on microbial community composition and ARG abundance when incorporated into functioning VFCWs has received considerably less attention.

Accordingly, this study aimed to evaluate how substrate configuration relates to antibiotic removal and microbial ecological profiles in laboratory-scale VFCWs exposed to OTC. We hypothesized that CoFe_2_O_4_ modification would not necessarily improve overall OTC removal, but would instead be associated with differences in OTC accumulation within the substrate, microbial community composition, and the abundance of tetracycline resistance genes and mobile genetic elements. By jointly considering pollutant removal and microbial ecological endpoints, this study provides a broader basis for evaluating substrate-assisted strategies for antibiotic resistance mitigation in constructed wetlands.

## 2. Materials and Methods

### 2.1. Preparation of CoFe_2_O_4_-Modified Biochar

CoFe_2_O_4_ was synthesized using a citrate-assisted calcination method adapted from previously reported citrate-based synthesis procedures [22]. Briefly, Co(NO_3_)_2_·6H_2_O and Fe(NO_3_)_3_·9H_2_O were dissolved in ultrapure water at a molar ratio of Co^2+^:Fe^3+^ = 1:2 and maintained at 60 °C under continuous stirring for 2 h. Citric acid (C_6_H_8_O_7_) was then added as a chelating agent at a molar concentration equivalent to the total metal-ion content, and the mixture was stirred at 60 °C for a further 2 h. Following complexation, the solution was dried under vacuum at 60 °C for 24 h to obtain a honeycomb-like precursor.

The dried precursor was ground in an agate mortar, transferred to a ceramic crucible, and calcined in a muffle furnace. The temperature was increased from room temperature to 400 °C at 10 °C min^−1^, held for 2 h, and then allowed to cool naturally, yielding black CoFe_2_O_4_ powder.

CoFe_2_O_4_-modified biochar was prepared by mixing the synthesized CoFe_2_O_4_ powder with crop-straw biochar (Changge CarbonNano Catalytic Technology Co., Ltd., Changge, China) at a loading of 5% (*w*/*w*). The mixture was homogenized by ball milling for 2 h to obtain the modified biochar used in subsequent experiments.

### 2.2. Characterization of CoFe_2_O_4_-Modified Biochar

The surface morphology of the pristine biochar and CoFe_2_O_4_-modified biochar was examined using scanning electron microscopy (SEM; Nova NanoSEM 450, FEI, Hillsboro, OR, USA). The specific surface area and pore characteristics were determined using an automated surface area and porosity analyzer (BSD-PS, BSD Instruments, Beijing, China). Specific surface area was calculated using the Brunauer–Emmett–Teller (BET) method over a relative pressure range of P/P_0_ = 0.05–1.00, while pore-size distribution was determined using the Barrett–Joyner–Halenda (BJH) model. The crystalline structure of the materials was characterized by X-ray diffraction (XRD; Empyrean, Malvern Panalytical, Almelo, The Netherlands), and diffraction peaks were identified by comparison with the ICDD PDF-4+ (2024) database.

### 2.3. Isothermal Adsorption and Kinetic Experiments

#### 2.3.1. Adsorption Kinetics

Adsorption kinetics were evaluated by adding 3.0 g of substrate to 100 mL of oxytetracycline (OTC) solution with an initial concentration of 100 mg L^−1^. The suspensions were incubated in a constant-temperature shaker at 20 °C and 150 rpm. Aliquots were collected after 5, 10, 20, 40, 80, 180, 360, 500 and 1020 min, and the residual OTC concentration was determined to calculate the adsorption capacity at time *t* (*Q_t_*). Adsorption kinetics were analyzed using the pseudo-first-order and pseudo-second-order kinetic models:

(1) $$\ln\left(Q_{e}-Q_{t}\right)=lnQ_{e}-k_{1}t$$

(2) $$\frac{t}{Q_{t}}=\frac{1}{k_{2}Q_{e}^{2}}+\frac{t}{Q_{e}}$$

where *Q_e_* (mg g^−1^) is the equilibrium adsorption capacity, *Q_t_* (mg g^−1^) is the adsorption capacity at time *t*, *k*_1_ (min^−1^) is the pseudo-first-order rate constant, and *k*_2_ (g mg^−1^ min^−1^) is the pseudo-second-order rate constant.

#### 2.3.2. Adsorption Isotherms

Adsorption isotherms were determined using OTC solutions with initial concentrations of 20, 40, 60, 80, 100, 120 and 140 mg L^−1^. The pH of each solution was adjusted to 7.0 using 0.1 M HCl or NaOH. Subsequently, 3.0 g of substrate was added to 100 mL of each solution, and the mixtures were equilibrated for 24 h at 20 °C and 150 rpm in a constant-temperature shaker. After equilibration, the supernatants were collected for OTC determination. The equilibrium adsorption data were fitted to the Langmuir and Freundlich isotherm models.

Langmuir model

(3) $$Q_{e}=\frac{Q_{m}K_{L}C_{e}}{1+K_{L}C_{e}}$$

Freundlich model

(4) $$Q_{e}=K_{F}C_{e}^{\frac{1}{n}}$$

where *Q_e_* (mg g^−1^) is the equilibrium adsorption capacity, *C_e_* (mg L^−1^) is the equilibrium concentration of OTC, *Q_m_* (mg g^−1^) is the theoretical maximum adsorption capacity, *K_L_* (L mg^−1^) and *K_F_* (mg^1−1/n^ L^1/n^ g^−1^) are the Langmuir and Freundlich adsorption constants, respectively, and n is the Freundlich heterogeneity factor. Each adsorption kinetics and isotherm experiment was performed in three independent replicates, and measured outcomes are expressed as mean ± SD.

### 2.4. Construction and Operation of Vertical Flow Constructed Wetlands

**Figure 1:**
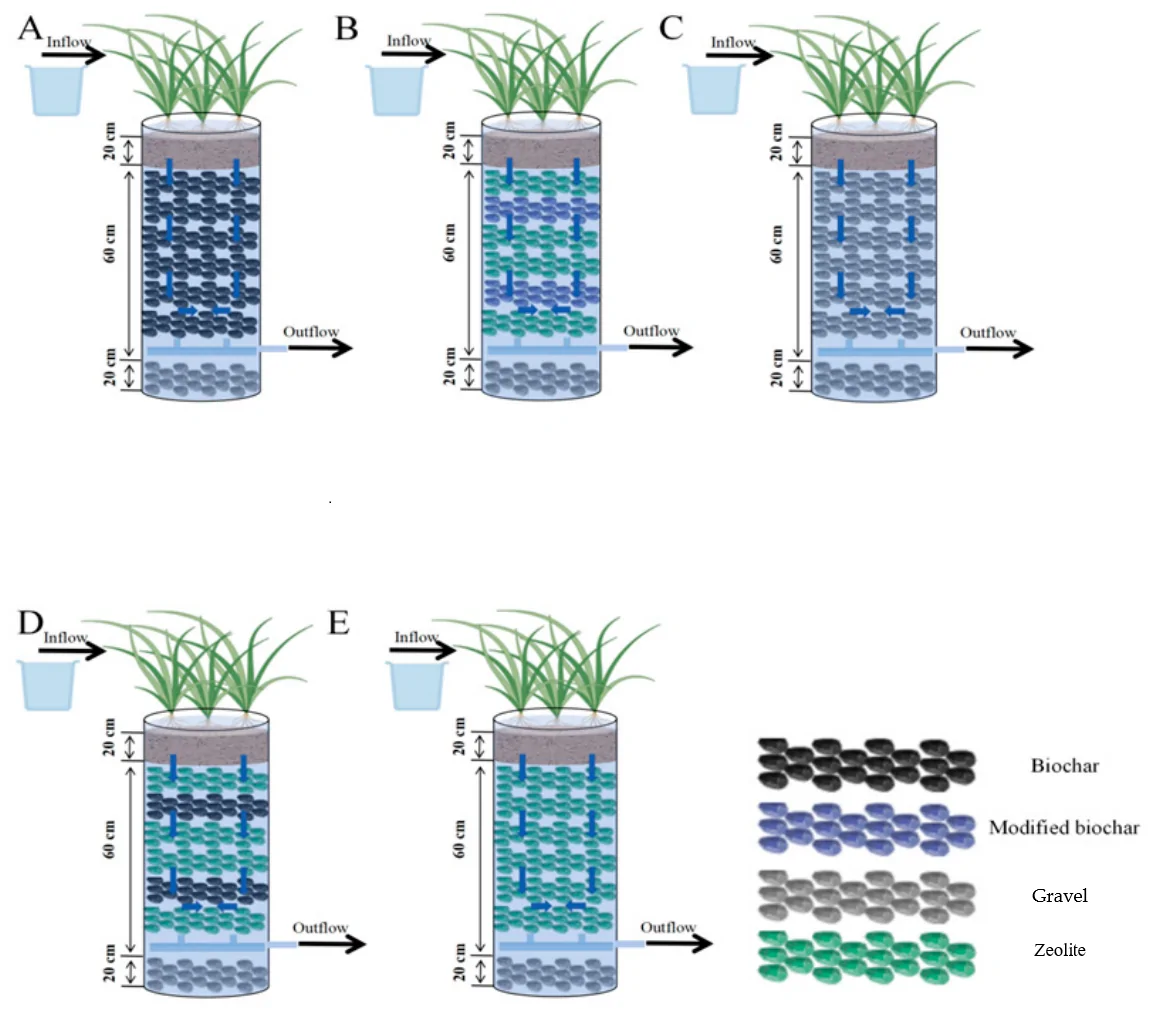
Schematic representation of the five laboratory-scale vertical flow constructed wetlands (VFCWs) used in this study. Each reactor represented one independent substrate treatment: (**A**) CW-C (biochar), (**B**) CW-GCF (CoFe_2_O_4_-modified biochar and zeolite, 1:2, *w*/*w*), (**C**) CW-LS (gravel), (**D**) CW-CF (biochar and zeolite, 1:2, *w*/*w*), and (**E**) CW-F (zeolite). All VFCWs consisted of a quartz sand distribution layer, a treatment-specific middle layer, and a gravel drainage layer, and were planted with cattails (*Typha* spp.). The blue arrows indicate the direction of water flow through the substrate layers.

Five laboratory-scale vertical flow constructed wetlands (VFCWs) (20 cm internal diameter × 100 cm height) were constructed, each representing one substrate treatment and constituting one independent experimental unit. Each VFCW consisted of three layers: (i) a 20-cm upper layer of 2–4 mm quartz sand serving as the water distribution and plant root-support layer; (ii) a 60-cm middle layer, which functioned as the principal treatment zone and contained the treatment-specific substrates; and (iii) a 20-cm lower layer of 10–15 mm gravel serving as the supporting and drainage layer. Prior to filling, all substrate materials were rinsed with ultrapure water to remove surface impurities. Five substrate configurations were evaluated: CW-LS (gravel), CW-F (zeolite), CW-C (biochar), CW-CF (biochar:zeolite, 1:2, *w*/*w*), and CW-GCF (CoFe_2_O_4_-modified biochar:zeolite, 1:2, *w*/*w*). All wetlands were planted with cattails (*Typha* spp.) at a density of 25 plants m^−2^ (Figure 1).

Following construction, all VFCWs were inoculated with anaerobic sludge obtained from the Zhengzhou Wastewater Treatment Plant (Zhengzhou, China) and acclimated for 20 days to establish microbial communities. Following a 20-day acclimation period, the wetlands were continuously fed with synthetic domestic wastewater amended with oxytetracycline (OTC). The experiment lasted 32 days and comprised three sequential phases with increasing OTC concentrations: 100 μg L^−1^ (Phase I, 7 days), 200 μg L^−1^ (Phase II, 7 days), and 500 μg L^−1^ (Phase III, 20 days). The first two phases were intended to acclimate the microbial communities to increasing antibiotic concentrations, whereas the final phase was used to evaluate treatment performance under sustained OTC exposure. During operation, all VFCWs were maintained at a hydraulic retention time of 2 days and operated outdoors under natural environmental conditions to better simulate practical wastewater treatment scenarios. At the end of the experimental period, substrate samples were collected from the upper, middle and lower layers of each VFCW for subsequent physicochemical and molecular analyses.

### 2.5. Preparation of Synthetic Domestic Wastewater

Synthetic domestic wastewater was prepared using tap water according to the formulation presented in Supplementary Table S1. Oxytetracycline was added to the influent at concentrations corresponding to the three operational phases of the experiment (100, 200 and 500 μg L^−1^), as described in Section 2.4. The use of synthetic wastewater enabled consistent influent composition throughout the experiment while simulating domestic wastewater supplemented with defined OTC concentrations.

### 2.6. Sample Collection and Preservation

At the end of the experimental period, substrate samples were collected separately from the upper (0–20 cm), middle (20–80 cm), and lower (80–100 cm) layers of each VFCW. At each substrate layer, three spatially separated subsamples were collected and homogenized to form one composite sample of approximately 20.0 ± 0.1 g. The three subsamples were used to improve the spatial representativeness of each composite sample and were not treated as independent experimental replicates. Samples were immediately transferred to sterile polyethylene bags, protected from light, stored at −20 °C, and processed within 24 h for physicochemical and molecular analyses.

Influent and effluent water samples were collected every 2 days throughout the experiment. Water samples were filtered through 0.22 μm membrane filters, transferred to 500 mL amber reagent bottles, transported to the laboratory under refrigerated conditions, and stored at 4 °C until analysis.

### 2.7. Determination of Oxytetracycline (OTC)

#### 2.7.1. Extraction of OTC from Substrate Samples

Oxytetracycline was extracted from freeze-dried substrate samples within 24 h of collection. Briefly, 10 g of freeze-dried substrate was mixed with 0.2 g Na_2_EDTA and 20 mL of an extraction solution consisting of phosphate buffer (pH 3.0) and acetonitrile (1:1, *v*/*v*). Samples were shaken for 20 min, sonicated for 10 min, and centrifuged to collect the supernatant. The extraction procedure was repeated three times, after which the combined extracts were diluted to 500 mL with ultrapure water.

#### 2.7.2. Extraction of OTC from Water Samples

Water samples (500 mL) were filtered through 0.45 μm membrane filters prior to solid-phase extraction (SPE). Oasis HLB cartridges were conditioned with 10 mL methanol followed by 10 mL ultrapure water. Samples were loaded at a flow rate of 10 mL min^−1^, after which cartridges were rinsed with 10 mL ultrapure water and dried under vacuum. Oxytetracycline was eluted with 6 mL methanol, and the eluates were evaporated under nitrogen for 20 min before being reconstituted in 2 mL of 20% methanol. The extracts were filtered through 0.22 μm membrane filters into amber vials. The pH was adjusted to 3.0 using 6 mol L^−1^ HCl, and 0.2 g EDTA was added to minimize interference from metal ions. Samples were stored at −20 °C until analysis.

#### 2.7.3. Instrumental Analysis

Oxytetracycline concentrations in environmental samples were determined using a Triple Quad™ 3500 LC-MS/MS system (SCIEX, Concord, ON, Canada). Chromatographic separation was performed using a mobile phase consisting of an aqueous phase [0.1% (*v*/*v*) formic acid in ultrapure water; A] and an organic phase [0.1% (*v*/*v*) formic acid in acetonitrile; B], using the gradient program shown in Supplementary Table S2. Mass spectrometric detection was performed in selected reaction monitoring (SRM) mode with an ion transfer tube temperature of 325 °C, ion source temperature of 350 °C, spray voltage of 4000 V, sheath gas pressure of 40 arb, auxiliary gas pressure of 10 arb, and collision gas pressure of 2 mTorr [24].

For the adsorption experiments, high OTC concentrations were quantified using a Thermo UltiMate 3000 HPLC (Thermo Fisher Scientific, Waltham, MA, USA) equipped with a Hypersil Gold C18 column (100 × 3.0 mm, 3 μm). The mobile phase consisted of an aqueous phase [0.1% (*v*/*v*) formic acid in ultrapure water; A] and an organic phase (acetonitrile; B), using the gradient program shown in Supplementary Table S2. Detection was performed at 254 nm, with a column temperature of 25 °C and an injection volume of 20 μL.

### 2.8. DNA Extraction and Quantitative Real-Time PCR (qPCR)

Genomic DNA was extracted from individual substrate samples collected from each layer of the VFCWs using the Ezup Soil DNA Purification Kit (Sangon Biotech, Shanghai, China) according to the manufacturer’s instructions. DNA concentration and purity were determined using a NanoDrop™ 2000 spectrophotometer (Thermo Fisher Scientific, Waltham, MA, USA). Purified DNA was stored at −80 °C until further analysis.

Quantitative real-time PCR (qPCR) was performed using a StepOnePlus™ Real-Time PCR System (Thermo Fisher Scientific, USA). The absolute abundance of the tetracycline resistance genes *tetA* and *tetX*, the mobile genetic elements *intI1*, *intI2*, and *ISCR1*, and the bacterial *16S rRNA* gene was quantified. Primer sequences for all target genes are provided in Supplementary Table S3 [25]. Each reaction contained 10 μL of 2× Taq Pro Universal SYBR qPCR Master Mix (Vazyme Biotech, Nanjing, China), 0.4 μM of each gene-specific forward and reverse primer, 2 μL of template DNA, and nuclease-free water to a final volume of 20 μL. Amplification was performed with an initial denaturation at 95 °C for 30 s, followed by 40 cycles of denaturation at 95 °C for 10 s and annealing/extension under the conditions specified in Supplementary Table S3. A melting curve analysis was performed at the end of each qPCR run to confirm amplification specificity. Each composite sample was analyzed using three technical qPCR replicates. These technical replicates were used to assess analytical reproducibility.

Absolute gene abundances (copies g^−1^) were determined using standard curves generated from purified plasmid standards constructed with the pMD19-T vector (TaKaRa Bio Inc., Shiga, Japan). Relative abundances were calculated as the ratio of ARG or MGE copy number to the corresponding *16S rRNA* gene copy number.

### 2.9. 16S rRNA Gene Amplicon Sequencing and Bioinformatics Analysis

For microbial community analysis, equal amounts of substrate collected from the upper, middle, and lower layers of each VFCW were homogenized to generate one composite sample representative of the entire wetland. Consequently, five composite samples (one per VFCW) were subjected to 16S rRNA gene amplicon sequencing.

The V3-V4 hypervariable region of the bacterial *16S rRNA* gene was amplified using primers 338F (5′-ACTCCTACGGGAGGCAGCA-3′) and 806R (5′-GGACTACHVGGTWTCTAAT-3′), as previously described [26]. Amplicon libraries were constructed and sequenced on an Illumina MiSeq platform (2 × 250 bp) by Sangon Biotech Co., Ltd. (Shanghai, China). Low-quality sequences (quality score < 20), primer mismatches, sequences shorter than 150 bp, and reads containing ambiguous bases were removed prior to clustering. Sequences were clustered into operational taxonomic units (OTUs) at 97% sequence similarity, and taxonomic assignment was performed against the SILVA reference database.

### 2.10. Statistical Analysis

Adsorption kinetic and isotherm models were fitted using OriginPro 2021 (OriginLab Corp., Northampton, MA, USA). Statistical analyses were performed using IBM SPSS Statistics 27.0 (IBM Corp., Armonk, NY, USA). For the VFCW experiment, each substrate configuration was represented by a single reactor. At each substrate layer, three spatially separated subsamples were pooled to form one composite sample before analysis. Because these samples and the corresponding analytical or technical replicates did not constitute independent reactor-level replicates, no inferential statistical comparisons of between-configuration effects were performed, and comparisons among VFCWs are reported descriptively.

Associations among environmental variables, ARGs, MGEs, and microbial taxa were explored using Spearman’s rank correlation coefficient. For the exploratory co-occurrence analysis, associations with a nominal *p* < 0.05 were retained for network visualization. Given the small number of composite microbial profiles (*n* = 5) and multiple pairwise comparisons, these associations were interpreted as exploratory rather than confirmatory.

Heatmaps showing the absolute and relative abundances of ARGs and MGEs were generated using GraphPad Prism 9.0 (GraphPad Software, San Diego, CA, USA). Differentially abundant microbial taxa were identified using linear discriminant analysis effect size (LEfSe) implemented on the BioinCloud platform v2.1.0. Microbial co-occurrence networks were constructed and visualized using Gephi 0.9 (The Gephi Consortium, Paris, France).

## 3. Results

### 3.1. CoFe_2_O_4_ Modification Altered the Physicochemical Properties of Biochar

**Figure 2:**
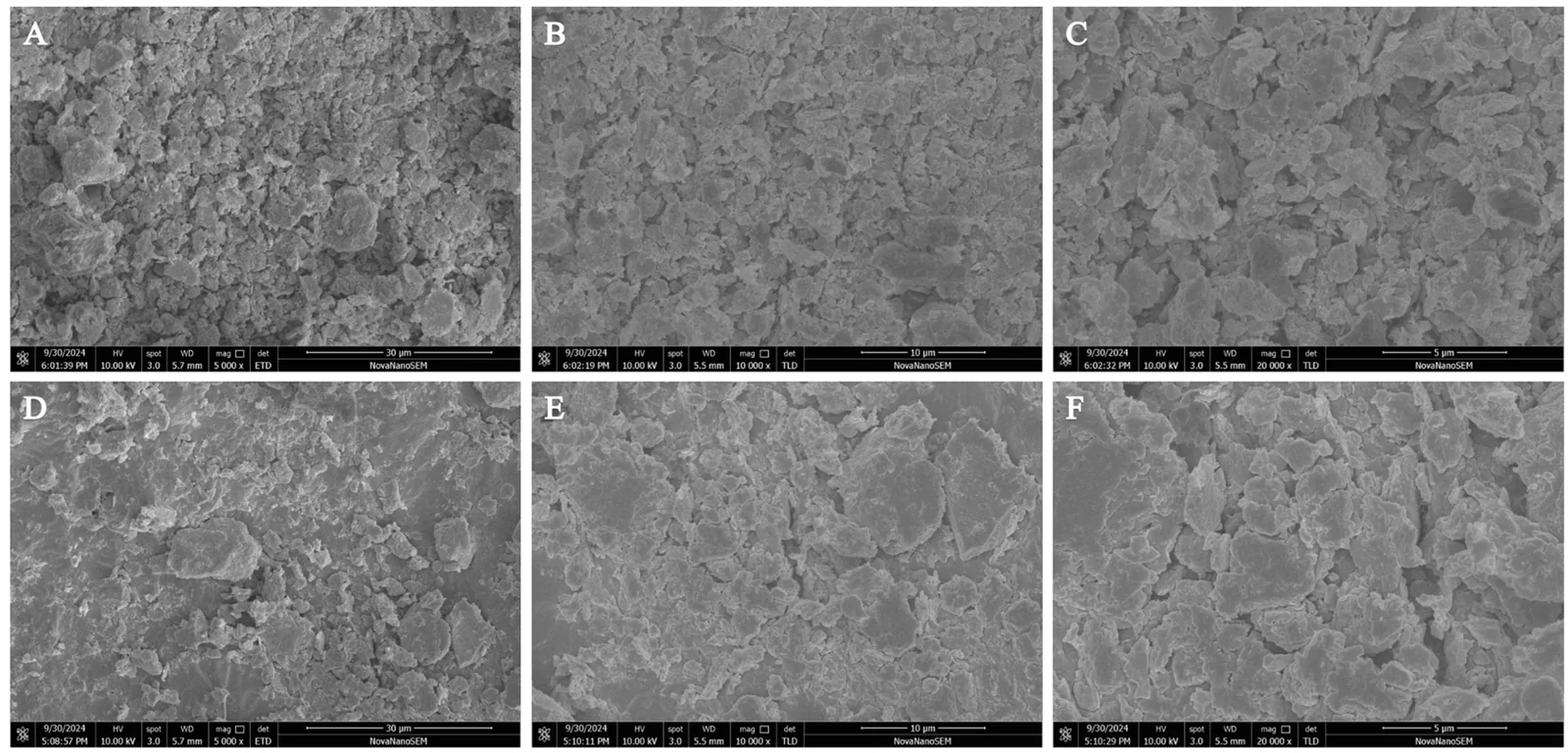
Scanning electron microscopy (SEM) images of pristine biochar (**A**–**C**) and CoFe_2_O_4_-modified biochar (**D**–**F**) at increasing magnifications.

Scanning electron microscopy (SEM) revealed clear morphological differences between pristine biochar and CoFe_2_O_4_-modified biochar (Figure 2). The unmodified biochar exhibited a relatively loose and porous structure composed of irregular flake-like particles with rough surfaces. Following CoFe_2_O_4_ modification, the surface was noticeably rougher, with a more heterogeneous distribution of smaller particles and increased aggregation of flake-like structures.

**Figure 3:**
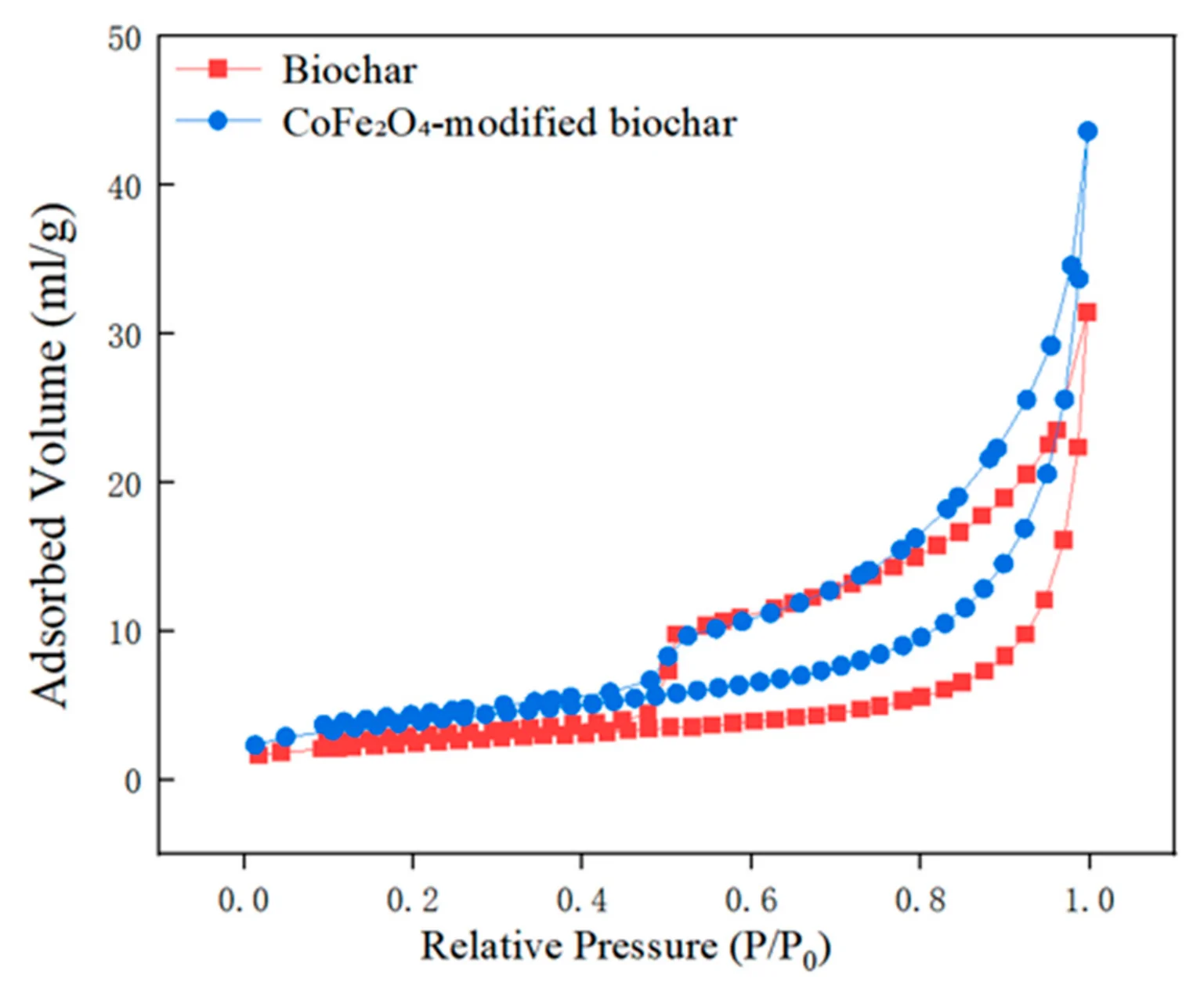
Nitrogen adsorption–desorption isotherms of pristine biochar and CoFe_2_O_4_-modified biochar.

**Figure 4:**
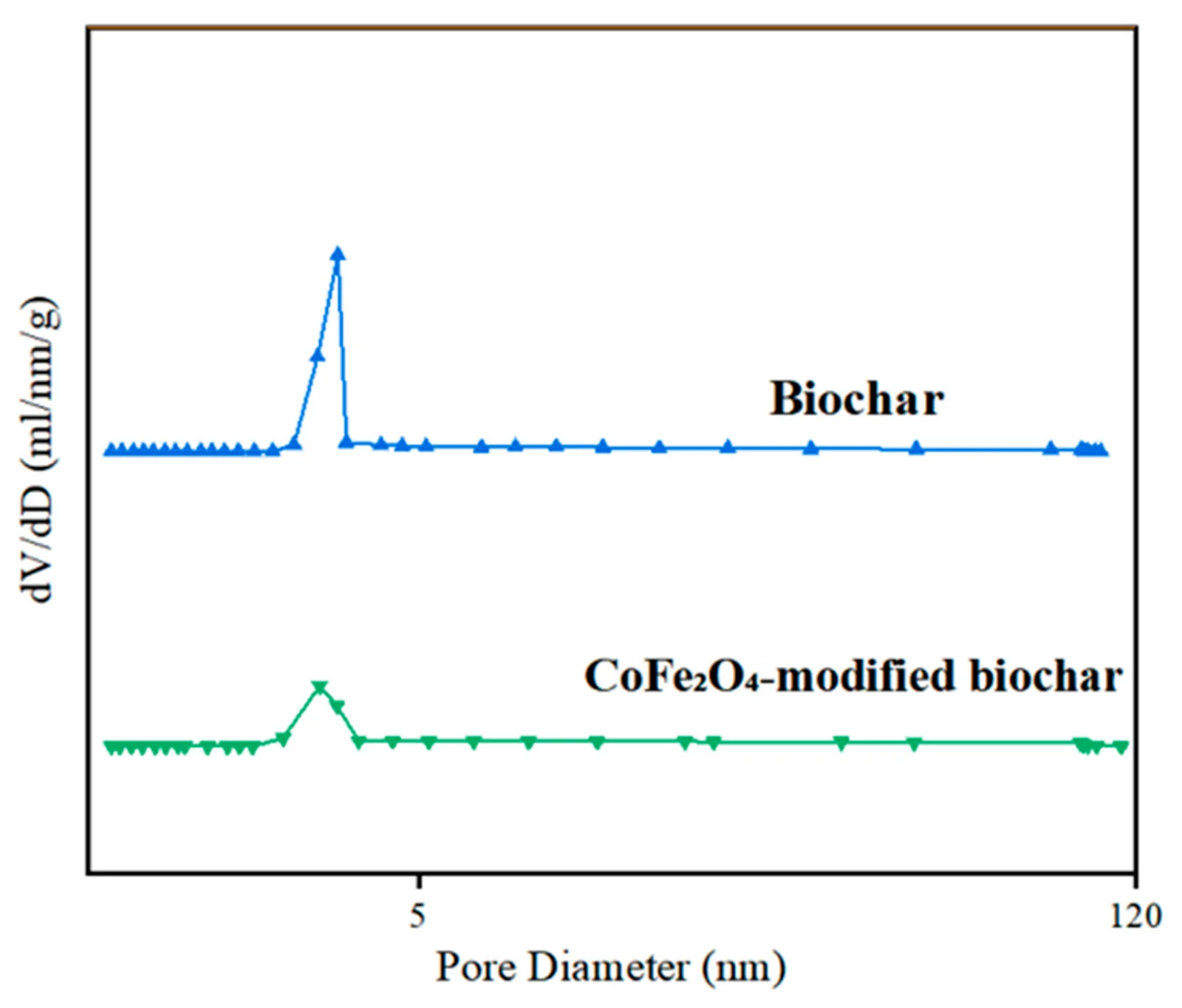
BJH pore-size distribution of pristine biochar and CoFe_2_O_4_-modified biochar.

Nitrogen adsorption–desorption isotherms and BJH pore-size distribution analyses further revealed differences in the pore characteristics between pristine and CoFe_2_O_4_-modified biochar (Figure 3 and Figure 4). Both materials exhibited type V adsorption isotherms with H3 hysteresis loops, indicating predominantly mesoporous structures. Compared with pristine biochar, the modified material exhibited higher nitrogen adsorption throughout the entire relative pressure range and a broader pore-size distribution with a slight shift towards smaller pore diameters.

These observations were supported by the quantitative BET analysis (Table 1). The CoFe_2_O_4_-modified biochar exhibited a specific surface area of 14.01 m^2^ g^−1^ and a total pore volume of 0.056 mL g^−1^, compared with 8.53 m^2^ g^−1^ and 0.040 m^2^ g^−1^, respectively, for pristine biochar, while the average pore diameter was 16.02 nm compared with 18.58 nm for pristine biochar.

### 3.2. CoFe_2_O_4_ Modification Exhibited Higher OTC Adsorption

**Figure 5:**
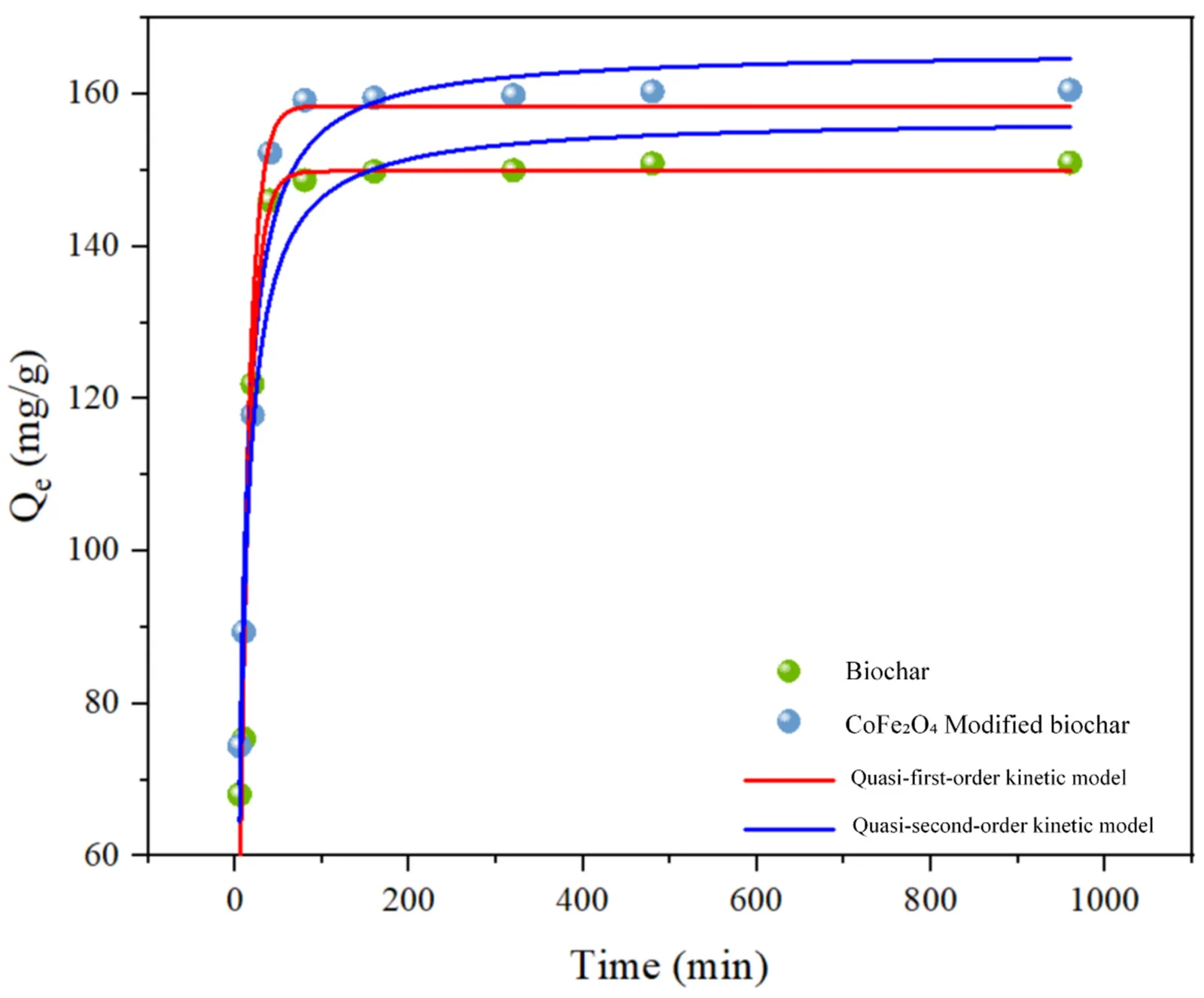
Adsorption kinetics of oxytetracycline (OTC) onto pristine biochar and CoFe_2_O_4_-modified biochar fitted using pseudo-first-order and pseudo-second-order kinetic models.

Adsorption kinetics showed that CoFe_2_O_4_-modified biochar had a higher equilibrium adsorption capacity for OTC than pristine biochar (Figure 5). Compared with pristine biochar, the modified material reached a higher equilibrium adsorption capacity while maintaining rapid adsorption during the initial stage of the experiment. Kinetic modelling showed that adsorption by pristine biochar was better described by the pseudo-first-order model (R^2^ = 0.953), whereas adsorption by CoFe_2_O_4_-modified biochar was better fitted by the pseudo-second-order model (R^2^ = 0.936), indicating that CoFe_2_O_4_ modification altered the adsorption behavior of the material.

**Figure 6:**
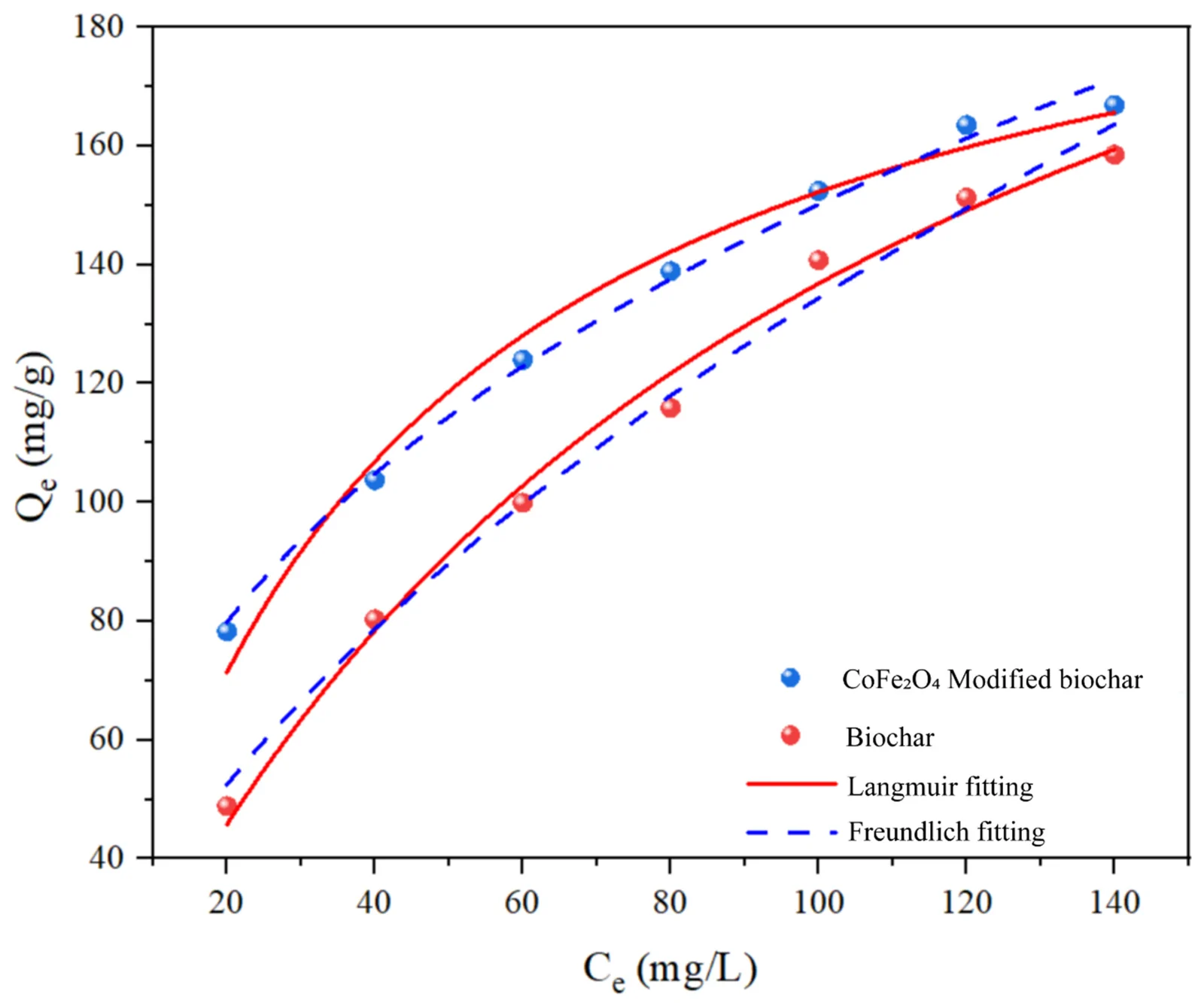
Adsorption isotherms of oxytetracycline (OTC) onto pristine biochar and CoFe_2_O_4_-modified biochar fitted using the Langmuir and Freundlich models.

The adsorption isotherms further confirmed the improved adsorption performance of the modified biochar (Figure 6). Across the entire range of equilibrium OTC concentrations, CoFe_2_O_4_-modified biochar consistently exhibited higher adsorption capacities than pristine biochar. Adsorption by pristine biochar was better described by the Langmuir model (R^2^ = 0.990), whereas adsorption by CoFe_2_O_4_-modified biochar showed a better fit to the Freundlich model (R^2^ = 0.993), consistent with a more heterogeneous adsorption pattern.

### 3.3. OTC Retention Varied Among VFCWs Despite Comparable Removal Efficiencies

**Figure 7:**
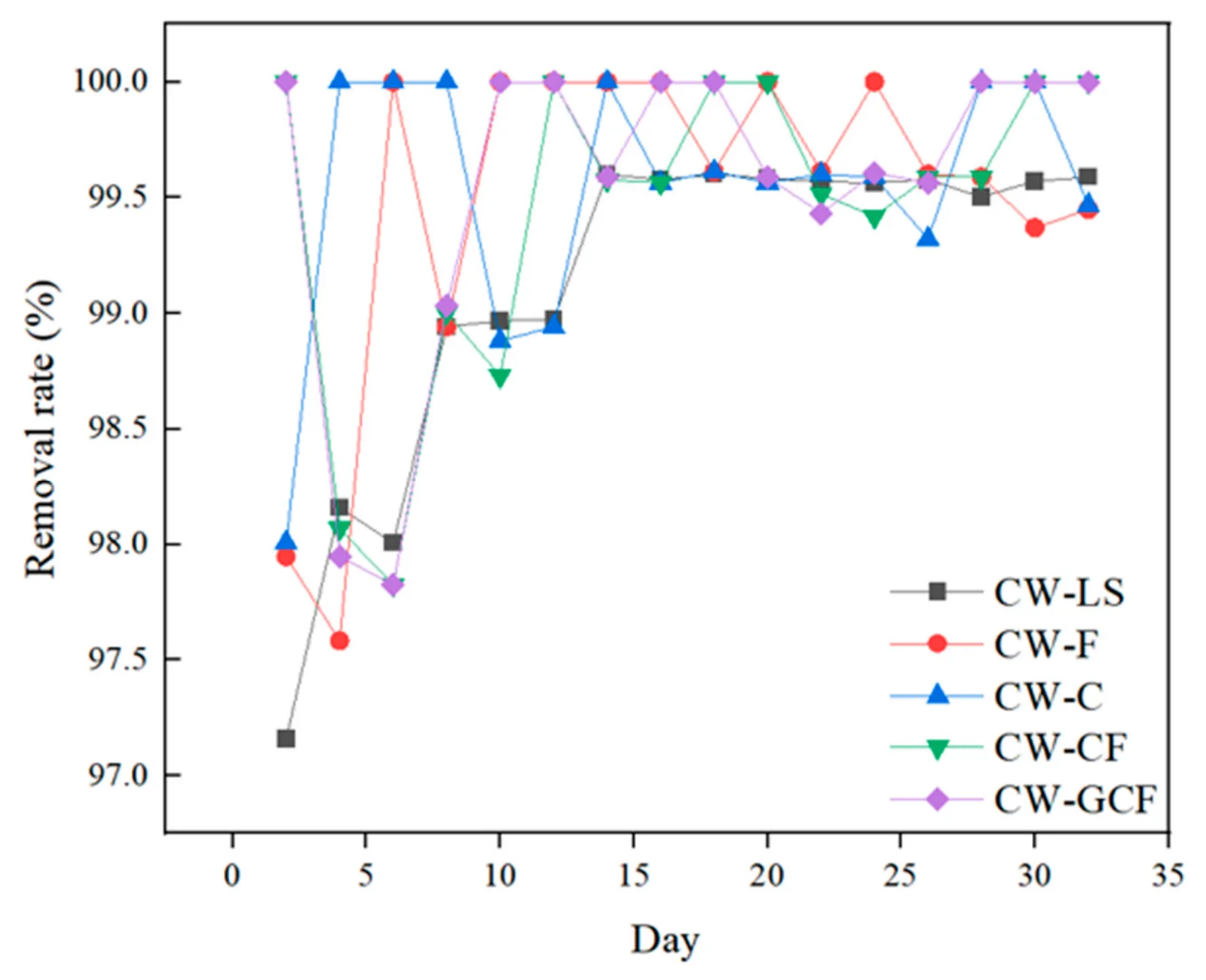
Oxytetracycline (OTC) removal efficiencies of the five vertical flow constructed wetlands (VFCWs) during the 32-day operational period. CW-LS (gravel); CW-F (zeolite); CW-C (biochar); CW-CF (biochar and zeolite, 1:2, *w*/*w*); CW-GCF (CoFe_2_O_4_-modified biochar and zeolite, 1:2, *w*/*w*).

All VFCWs maintained consistently high OTC removal efficiencies throughout the 32-day operational period (Figure 7). Removal rates remained above 97.16% in all systems, with an overall average of 99.43 ± 0.15%. No substantial differences in effluent OTC removal were observed among the five substrate configurations.

**Figure 8:**
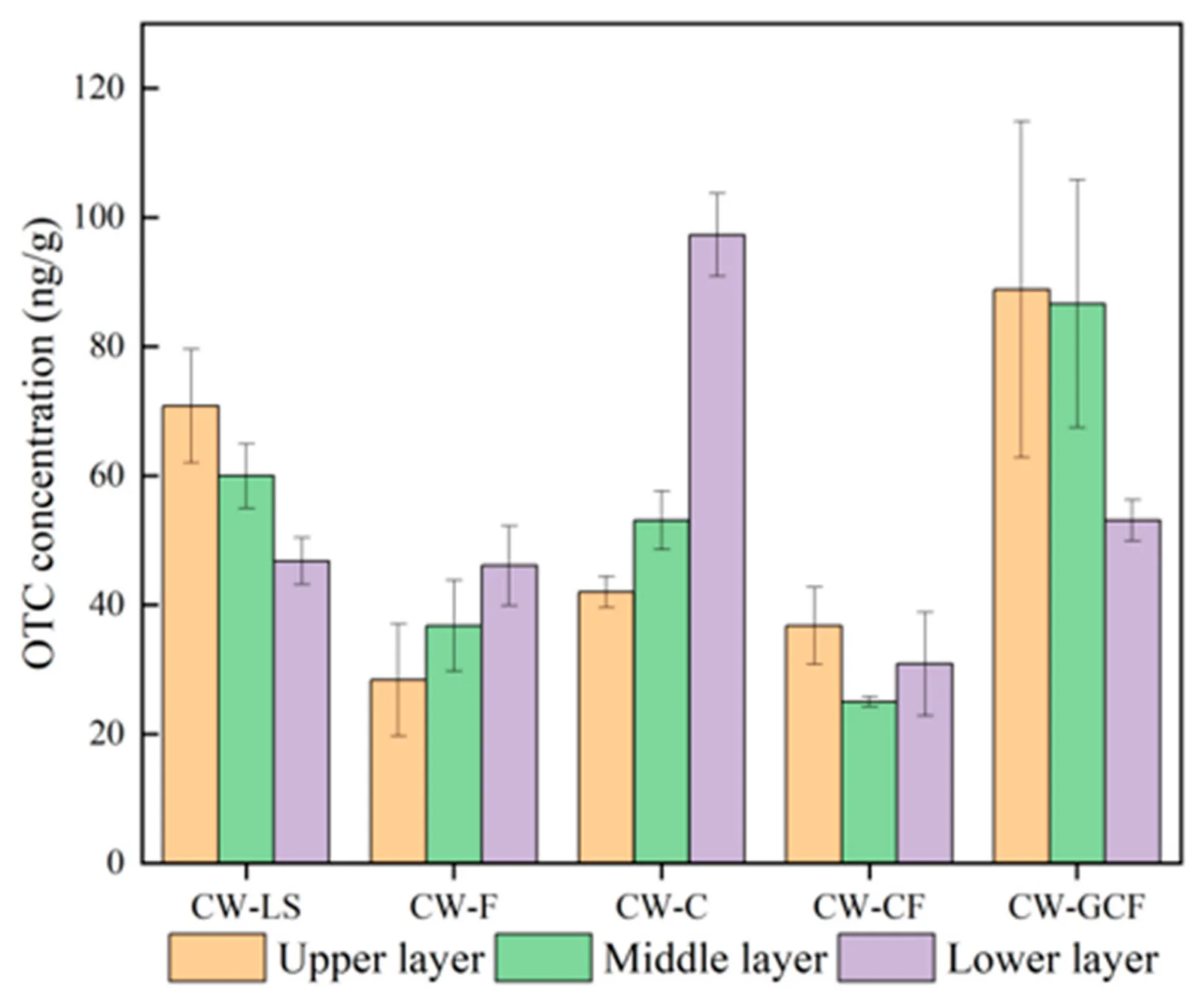
Oxytetracycline (OTC) concentrations in the upper, middle, and lower substrate layers of the five VFCWs at the end of the experiment. CW-LS (gravel); CW-F (zeolite); CW-C (biochar); CW-CF (biochar and zeolite, 1:2, *w*/*w*); CW-GCF (CoFe_2_O_4_-modified biochar and zeolite, 1:2, *w*/*w*).

Despite these comparable removal efficiencies, marked differences were observed in OTC accumulation within the wetland substrates (Figure 8). Overall, the CoFe_2_O_4_-modified biochar–zeolite exhibited the highest mean OTC accumulation, followed by the biochar reactor, whereas the biochar–zeolite reactor showed the lowest accumulation. The spatial distribution of OTC also differed among reactors. In CoFe_2_O_4_-modified biochar–zeolite, the highest OTC concentrations occurred in the upper and middle substrate layers, whereas in biochar the greatest accumulation was detected in the lower layer. These results indicate that although similar OTC removal from the aqueous phase was observed across the different substrate configurations, OTC retention within the wetland matrix differed among reactors.

### 3.4. Antibiotic Resistance Gene Profiles Varied Among VFCWs

**Figure 9:**
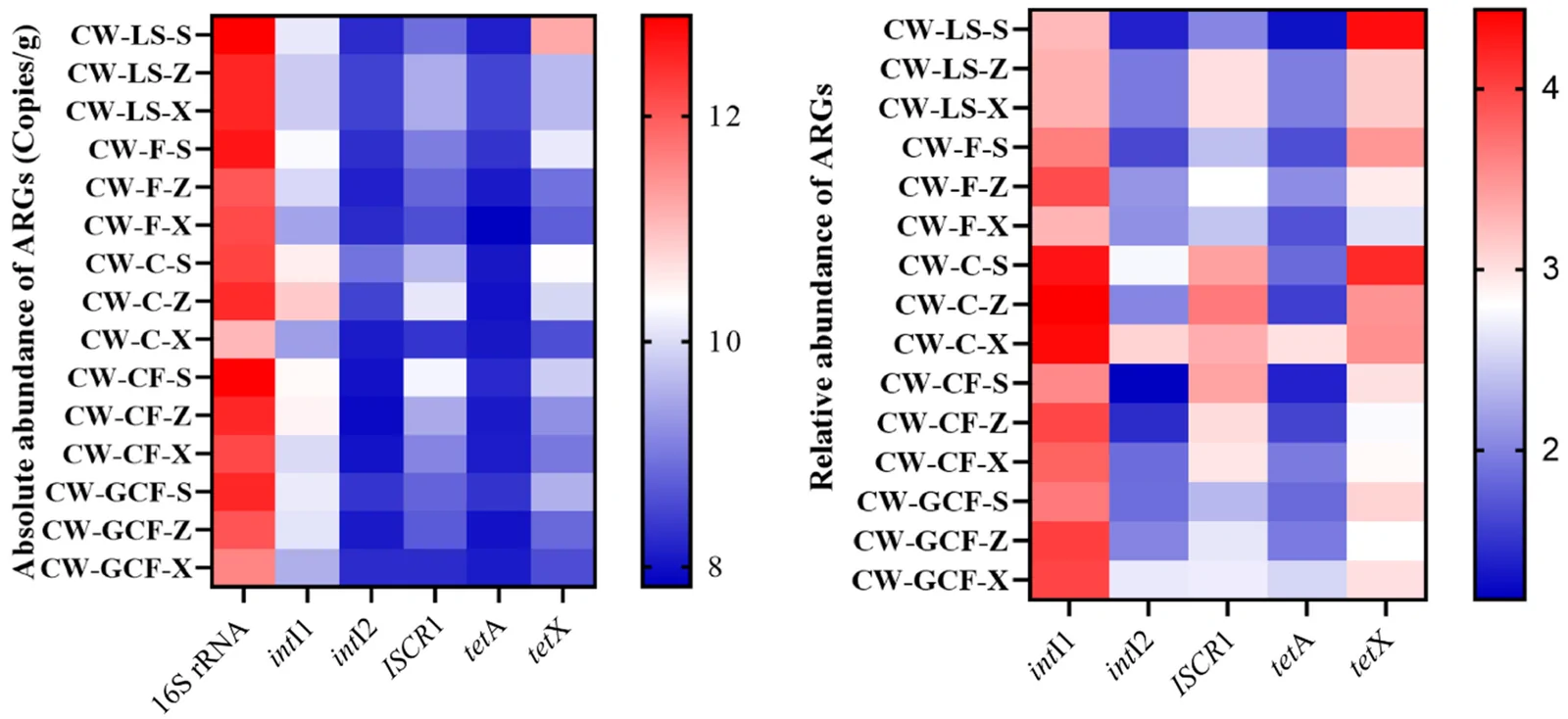
Heatmaps showing the absolute (**left**) and relative (**right**) abundances of tetracycline resistance *tetA* and *tetX* genes, mobile genetic elements (*intI1*, *intI2*, and *ISCR1*), and the bacterial *16S rRNA* gene in the upper (S), middle (Z), and lower (X) substrate layers of the five VFCWs: CW-LS (gravel); CW-F (zeolite); CW-C (biochar); CW-CF (biochar and zeolite, 1:2, *w*/*w*); CW-GCF (CoFe_2_O_4_-modified biochar and zeolite, 1:2, *w*/*w*).

The abundances of the bacterial *16S rRNA* gene, tetracycline resistance *tetA* and *tetX* genes, and mobile genetic elements *intI1*, *intI2*, and *ISCR1* were quantified in the upper, middle, and lower substrate layers of each VFCW (Figure 9). The *16S rRNA* gene abundance ranged from 1.17 × 10^5^ to 7.73 × 10^6^ copies g^−1^, with the highest values generally observed in the upper substrate layer. The distribution of the three MGEs followed a similar vertical pattern, with *intI1* consistently exhibiting higher abundance than *intI2* and *ISCR1*.

The total abundance of *tetA* and *tetX* genes ranged from 2.55 × 10^2^ to 1.90 × 10^5^ copies g^−1^ and was highest in the upper substrate layer across all VFCWs. ARG profiles varied among reactors. The mean absolute ARG abundance decreased in the order gravel > biochar > zeolite > biochar–zeolite > CoFe_2_O_4_-modified biochar–zeolite, whereas the mean relative abundance decreased in the order gravel > biochar > zeolite > CoFe_2_O_4_-modified biochar–zeolite > biochar–zeolite.

### 3.5. Microbial Community Profiles Varied Among VFCWs

High-throughput sequencing of the bacterial *16S rRNA* gene generated 384,863 high-quality sequences across the five VFCWs. Taxonomic annotation identified 36 bacterial phyla and 745 genera, indicating a highly diverse microbial community within the wetland substrates. Descriptive alpha-diversity values varied among the five composite profiles (Table 2). The biochar reactor exhibited the highest richness (Chao1 = 1701.3), Shannon diversity (8.41), and phylogenetic diversity (PD = 37.64), whereas the gravel reactor displayed the lowest corresponding values (821.8, 5.30, 24.08, respectively). Sequencing coverage exceeded 99.9% for all samples, indicating that the sequencing depth was sufficient to characterize the bacterial communities. Because one composite sample was sequenced per reactor, these values characterize individual reactors and do not establish treatment-level differences.

**Figure 10:**
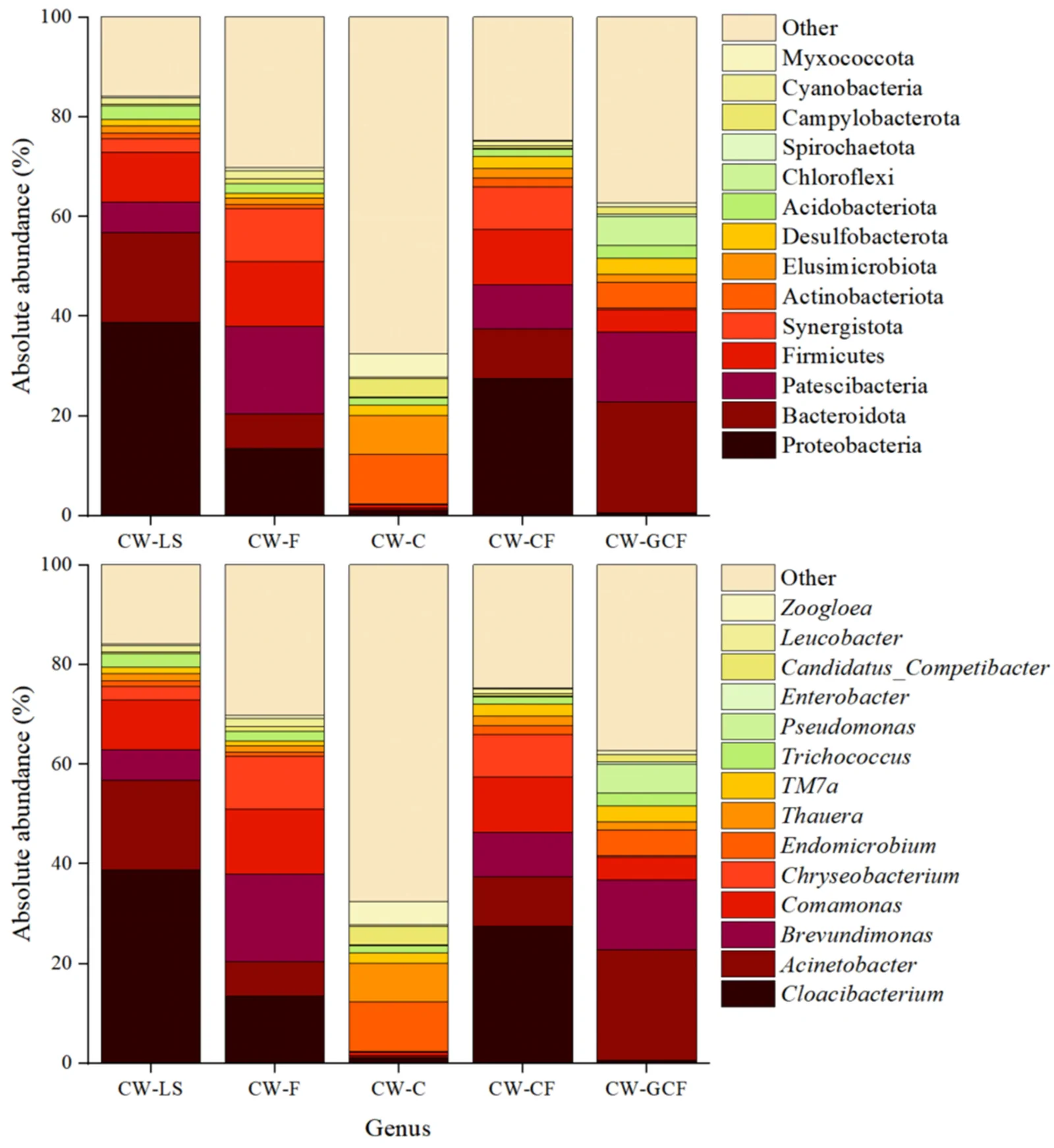
Bacterial community composition of the five VFCWs at the phylum (upper panel) and genus (lower panel) levels based on *16S rRNA* gene amplicon sequencing. Only the most abundant taxa are shown; remaining taxa are grouped as “Other”. CW-LS (gravel); CW-F (zeolite); CW-C (biochar); CW-CF (biochar and zeolite, 1:2, *w*/*w*); CW-GCF (CoFe_2_O_4_-modified biochar and zeolite, 1:2, *w*/*w*).

The bacterial community composition also differed among five reactor profiles (Figure 10). At the phylum level, the communities were dominated by Proteobacteria, Bacteroidota, and Patescibacteria, although their relative abundances varied among reactors. At the genus level, the relative abundances of dominant genera also varied among reactors, with *Cloacibacterium*, *Acinetobacter*, and *Endomicrobium* among the prominent genera.

**Figure 11:**
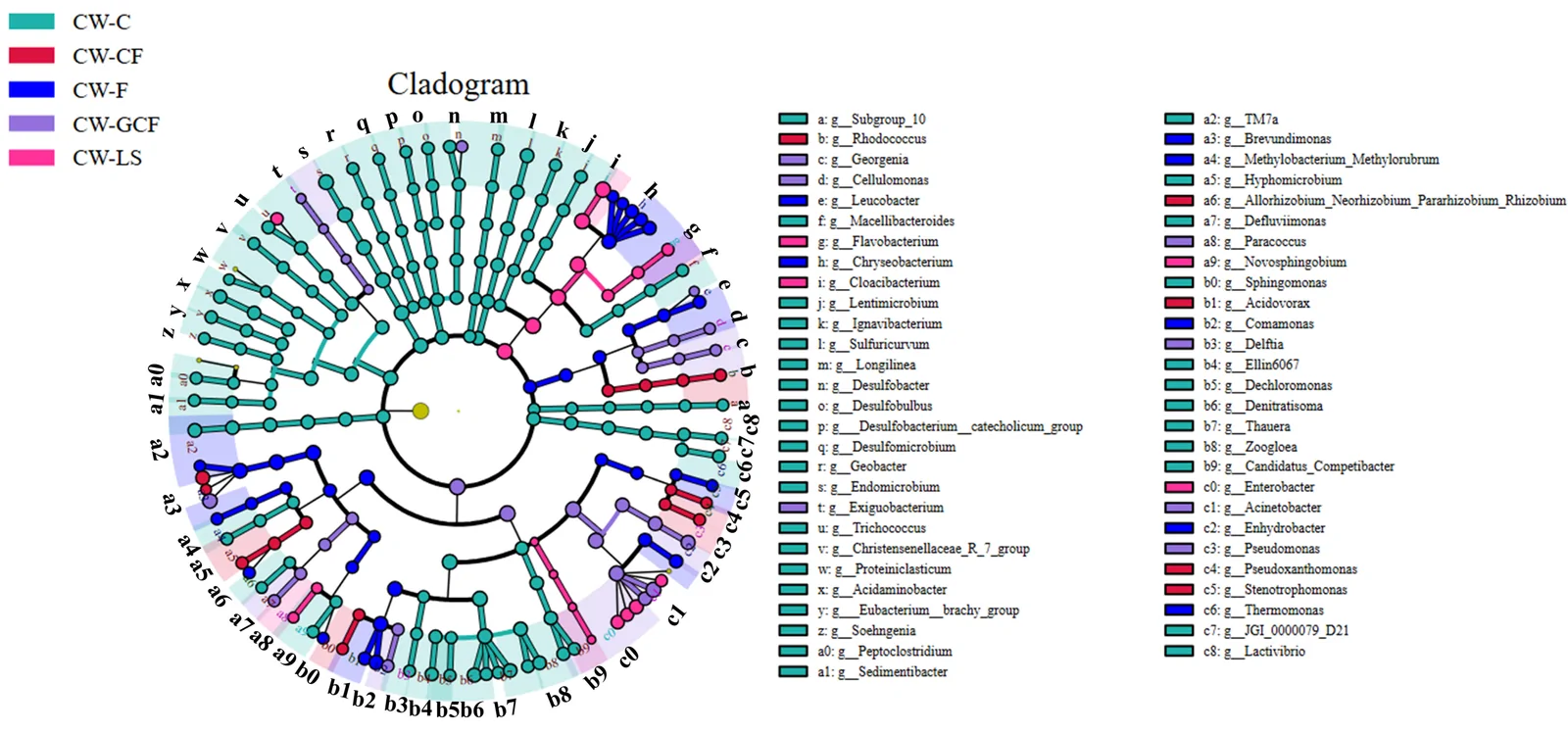
Exploratory LEfSe cladogram highlighting bacterial taxa contributing to differentiation among the five VFCW reactors. Colours indicate the reactor in which each taxon contributed most strongly to differentiation: CW-C (turquoise), CW-CF (red), CW-F (blue), CW-GCF (purple), and CW-LS (pink). Circles represent taxa at successive taxonomic levels from the centre towards the periphery of the cladogram, and circle size is proportional to relative abundance. Yellow nodes indicate taxa not assigned to a specific reactor. Lowercase letter codes (a–c8) positioned around the cladogram correspond to the bacterial genera listed in the accompanying key. Because only one composite microbial sample was analysed per reactor, these features are presented descriptively and should not be interpreted as statistically validated treatment biomarkers or evidence of treatment-specific enrichment.

Exploratory LEfSe analysis highlighted taxa contributing to differentiation among individual reactor profiles (Figure 11). Proteobacteria was prominent in the CoFe_2_O_4_-modified biochar–zeolite profile, Bacteroidota in the gravel profile, and Patescibacteria in the biochar profile. Because only one composite sample was available per reactor, these patterns are presented descriptively and do not establish treatment-specific enrichment.

### 3.6. Co-Occurrence Network Analysis Revealed Potential Associations Between ARGs, MGEs, and Bacterial Taxa

**Figure 12:**
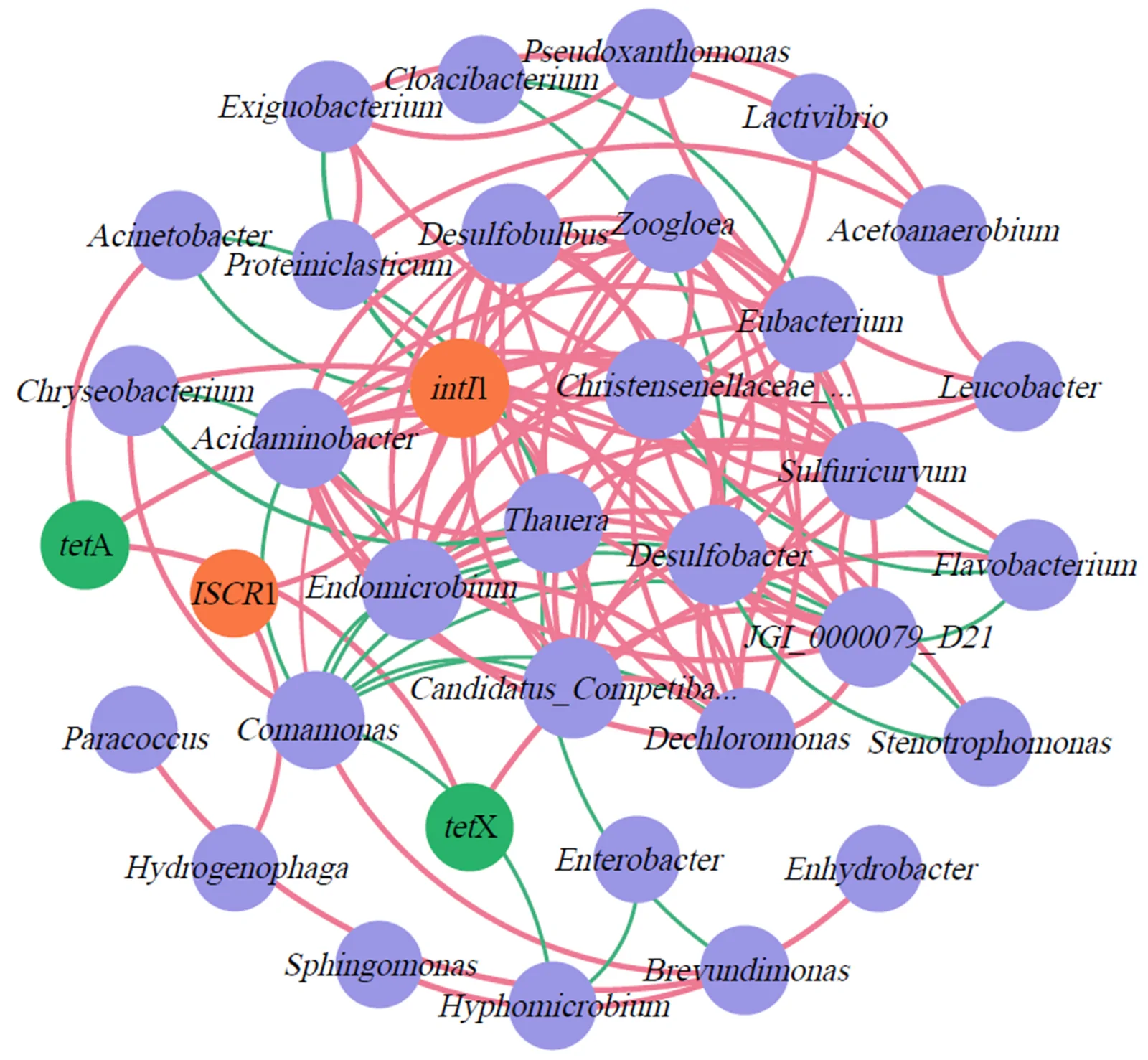
Co-occurrence network showing associations meeting the nominal Spearman criterion (*p* < 0.05) among tetracycline resistance genes (*tetA* and *tetX*), mobile genetic elements (MGEs), and the most abundant bacterial genera across the five composite microbial profiles. Red edges indicate positive correlations, whereas green edges indicate negative correlations. Associations are exploratory and indicate covariation only; they do not establish ARG host identity or horizontal gene transfer.

A co-occurrence network was constructed using *tetA* and *tetX* genes, the mobile genetic elements *intI1*, *intI2*, and *ISCR1*, and the most abundant bacterial genera (top 1% relative abundance) to explore potential associations among these components (Figure 12). The resulting network comprised 32 nodes and 99 correlations meeting the nominal Spearman criterion (*p* < 0.05), with a modularity value of 0.46. Given the small number of composite samples (*n* = 5) and multiple pairwise comparisons, these correlations were treated as exploratory associations.

Among the MGEs, *intI1* showed positive associations with several bacterial genera, including *Thauera* and *Endomicrobium*, indicating covariation between *intI1* abundance and the relative abundances of these genera across the five composite profiles. *Acinetobacter* showed a positive association with *tetA* and a negative association with *intI1*, whereas *Flavobacterium* was positively associated with both *tetA* and *tetX*. In addition, *tetA* and *tetX* were positively correlated with each other. These associations represent exploratory covariation only; they neither demonstrate that the associated bacterial genera carry *tetA* or *tetX* nor provide direct evidence of horizontal gene transfer.

## 4. Discussion

Constructed wetlands are increasingly recognized as sustainable technologies for mitigating antibiotic contamination in wastewater [11,13,27,28]. However, their environmental performance should not be evaluated solely on the basis of contaminant removal efficiency, because microbial ecological processes occurring within wetland substrates may influence the persistence and dissemination of antibiotic resistance. In the present study, all five VFCWs achieved consistently high OTC removal efficiencies (>97%). Nevertheless, marked differences were observed among reactors in OTC accumulation within the substrate matrix, ARG and MGE abundances, and microbial community composition, with the CoFe_2_O_4_-modified biochar–zeolite reactor exhibiting the greatest OTC retention and the lowest mean ARG abundance. These observations illustrate that VFCWs with similarly high pollutant removal efficiencies may nevertheless exhibit contrasting ARG and microbial community profiles, supporting the view that pollutant removal and microbial ecological endpoints should be considered complementary dimensions of constructed wetland performance [29]. The observed patterns therefore provide a basis for exploring how substrate properties may be associated with antibiotic retention and the microbial ecology of antibiotic resistance in constructed wetlands.

Although greater OTC accumulation was observed in the CoFe_2_O_4_-modified biochar–zeolite reactor, particularly in the upper and middle layers, this was not accompanied by greater OTC removal from the effluent. One possible explanation for this apparent discrepancy is the contribution of complementary mechanisms involved in antibiotic attenuation in constructed wetlands. Previous studies have shown that adsorption operates alongside microbial biodegradation, plant uptake, photodegradation, and other abiotic transformation processes, with the relative contribution of each pathway depending on substrate properties and wetland operating conditions [9,15,30]. Thus, concurrent removal processes may have contributed to the similarly high overall treatment efficiencies observed across the five reactors despite differences in substrate OTC accumulation. The independently replicated batch experiments provide direct evidence that CoFe_2_O_4_ modification altered the adsorption properties of biochar. CoFe_2_O_4_ modification increased the specific surface area and pore volume of the biochar and shifted the adsorption behaviour from predominantly Langmuir-type to Freundlich-type, consistent with increased surface heterogeneity and a broader distribution of adsorption sites. Similar changes have been reported following metal-oxide modification of biochar, where increased surface complexity can enhance pollutant retention through additional adsorption sites and heterogeneous adsorption mechanisms [31,32]. Beyond adsorption, modified substrates may also alter the physicochemical microenvironment associated with microbial community structure, microbial activity, and pollutant transformation within constructed wetlands, as reported in previous studies [33].

Differences among reactors were also evident in the observed abundances of ARGs and MGEs. Despite similarly high OTC removal efficiencies across all five VFCWs, ARG and MGE profiles varied among reactors. The CoFe_2_O_4_-modified biochar–zeolite reactor exhibited the lowest mean ARG abundance, whereas the conventional gravel reactor showed the highest. A decoupling between antibiotic removal and ARG attenuation has also been reported in wastewater treatment systems and constructed wetlands [4,5,13]. The pattern observed here is consistent with this concept and suggests that conventional contaminant-removal metrics alone may not capture differences in ARG profiles within treatment systems, supporting the consideration of microbial ecological endpoints as complementary indicators of wetland performance. Antibiotic removal and ARG persistence may reflect partly different processes: whereas antibiotic removal reflects contaminant attenuation, ARG persistence can be associated with factors including antibiotic selection pressure, microbial community structure, and horizontal gene transfer [4,34]. These processes may respond differently to substrate properties and operating conditions than contaminant removal itself. Consequently, high antibiotic removal efficiency does not necessarily imply a correspondingly low ARG abundance, emphasizing the value of evaluating both endpoints when assessing constructed wetland performance.

Several mechanisms could potentially contribute to the lower ARG abundance observed in the CoFe_2_O_4_-modified biochar–zeolite reactor. These mechanisms may involve changes in antibiotic bioavailability, microbial community structure, and ecological interactions that affect the conditions under which ARGs are maintained and disseminated. One possibility is that the greater adsorption capacity of the modified biochar reduced the bioavailable fraction of OTC within the substrate, potentially lowering antibiotic selection pressure despite the greater total OTC accumulation observed in this reactor. This hypothesis is consistent with previous studies showing that modified biochar can enhance antibiotic adsorption and decrease contaminant bioavailability through stronger sorption interactions [31]. In addition, biochar-based materials have been reported to adsorb extracellular DNA, which could reduce the persistence of extracellular ARGs and opportunities for horizontal gene transfer [35]. CoFe_2_O_4_ has also been reported to generate reactive oxygen species under suitable environmental conditions, potentially affecting bacterial activity and ARG persistence [20]. These mechanisms provide biologically plausible explanations for the lower ARG and MGE abundance observed in the CoFe_2_O_4_-modified biochar–zeolite reactor. However, antibiotic bioavailability, extracellular DNA adsorption, reactive oxygen species generation, and horizontal gene transfer were not directly measured in the present study. Accordingly, these explanations should be regarded as testable hypotheses rather than experimentally demonstrated mechanisms.

The lower ARG abundance observed in the CoFe_2_O_4_-modified biochar–zeolite reactor coincided with differences in microbial community composition relative to the other reactors. Previous studies have shown that substrate physicochemical properties, including porosity, surface chemistry, and oxygen availability, can influence microbial colonization and biofilm development, thereby affecting microbial processes relevant to constructed wetland performance [36,37]. In the present study, differences in bacterial community profiles were observed among reactors despite their operation under the same hydraulic conditions and exposure to the same influent. Notably, the CoFe_2_O_4_-modified biochar–zeolite reactor, which exhibited the lowest ARG abundance, also showed a bacterial community profile that differed from those of the other reactors. This co-occurrence is consistent with a potential relationship between microbial community composition and ARG abundance; however, given the lack of independent reactor-level replication, it cannot establish that substrate composition drove microbial community assembly or the observed ARG profile.

The dominant bacterial phyla identified in this study, including Proteobacteria, Bacteroidota, and Patescibacteria, are widely recognized as key members of microbial communities associated with constructed wetlands and wastewater treatment systems. Proteobacteria are metabolically versatile microorganisms involved in carbon, nitrogen, and organic pollutant transformation, but they also represent important environmental reservoirs of ARGs and MGEs [38]. Likewise, members of Bacteroidota possess intrinsic and acquired resistance mechanisms and have been implicated in the horizontal transfer of ARGs under antibiotic selection pressure [39]. In contrast, Patescibacteria are characterized by streamlined genomes and extensive ecological dependence on neighboring microorganisms, with increasing evidence indicating that horizontal gene transfer plays an important role in their evolution and adaptation [40,41]. Although the present data do not establish direct causal relationships between individual taxa and ARG dissemination, the observed differences in the relative abundance of these dominant phyla provide ecological context for the variation in ARG profiles among reactors. Whether substrate properties contributed to these patterns and through which mechanisms cannot be determined from the present experiment.

Exploratory LEfSe and co-occurrence analyses provided complementary descriptive perspectives on microbial community variation across the five composite profiles. LEfSe highlighted taxa contributing to differentiation among individual reactor profiles; however, because only one composite sample was sequenced per reactor, these features should be interpreted as profile-associated rather than as statistically validated treatment biomarkers. Exploratory co-occurrence analysis further identified associations among bacterial genera, ARGs, and MGEs meeting the nominal Spearman criterion (*p* < 0.05). Among these, positive associations involving *intI1*, *tetA*, and *tetX* are biologically noteworthy because class 1 integrons are widely recognized as important elements associated with the capture and dissemination of ARGs in environmental microbial communities [42]. Nevertheless, given the small number of composite profiles (n = 5), the multiple pairwise comparisons, and the absence of direct measurements of ARG host identity or horizontal gene transfer, these associations should be regarded as exploratory covariation and do not establish ecological interactions, ARG mobility, or mechanisms of resistance dissemination.

Taken together, the present findings highlight the value of considering microbial ecological endpoints alongside conventional contaminant-removal metrics when evaluating constructed wetland performance. Antibiotic removal efficiency alone may provide an incomplete picture, as reactors exhibiting similarly high OTC removal showed contrasting ARG and MGE abundances and microbial community profiles. Incorporating endpoints such as ARG abundance, MGE abundance, and microbial community composition may therefore provide a more comprehensive assessment of the microbial ecological context associated with treatment performance [29,34,37]. An important limitation of the present study is that each substrate configuration was represented by a single VFCW reactor. Consequently, the observed differences among reactors cannot be interpreted as statistically established treatment effects, and the findings should be considered exploratory and hypothesis-generating. Independent reactor-level replication will be required to determine whether the observed patterns are reproducible across substrate configurations. In addition, the proposed mechanisms linking substrate properties, antibiotic bioavailability, microbial community structure, and ARG abundance were not directly tested and should therefore be regarded as hypotheses for future investigation. Future studies combining independently replicated wetland systems with direct measurements of antibiotic bioavailability and microbial selection pressure, together with metagenomics, metatranscriptomics, and long-term field validation, could clarify the functional relationships underlying the patterns observed here. Such studies would help determine whether substrate engineering can be used to simultaneously maintain efficient antibiotic removal and reduce conditions associated with the persistence and dissemination of antibiotic resistance in constructed wetlands.

## Figures and Tables

**Table 1:** BET surface area and pore characteristics of pristine biochar and CoFe_2_O_4_-modified biochar.

Sample	BET Surface Area (m^2^ g^−1^)	Total Pore Volume (mL g^−1^)	Average Pore Diameter (nm)
Biochar	8.53	0.040	18.58
CoFe_2_O_4_-modified biochar	14.01	0.056	16.02

**Table 2:** Alpha-diversity indices of bacterial communities in the five VFCWs ^1^, based on 16S rRNA gene amplicon sequencing.

Sample ID	Feature	Chao1	Simpson	Shannon	PD_Whole_Tree	Coverage
CW-LS	821	821.8	0.88	5.30	24.08	0.9996
CW-F	1112	1112.6	0.96	6.56	30.81	0.9997
CW-C	1701	1701.3	0.99	8.41	37.64	0.9996
CW-CF	876	876.6	0.94	6.13	27.70	0.9997
CW-GCF	1250	1250.9	0.96	6.86	32.81	0.9996
Total	5760	5763.2	4.73	33.27	153.05	4.9982

^1^ CW-LS (gravel); CW-F (zeolite); CW-C (biochar); CW-CF (biochar and zeolite, 1:2, *w*/*w*); CW-GCF (CoFe_2_O_4_-modified biochar and zeolite, 1:2, *w*/*w*).

## Data Availability

The original contributions presented in this study are included in the article/Supplementary Materials. Further inquiries can be directed to the corresponding authors.

## Supplementary Materials

The following supporting information can be downloaded at: https://www.mdpi.com/article/10.3390/microorganisms14091950/s1, Table S1: Composition of the synthetic domestic wastewater used throughout the vertical flow constructed wetland experiment; Table S2: Mobile phase gradient elution program; Table S3: qPCR Primer sequences, resistance mechanisms, and annealing temperatures.

## Abbreviations

The following abbreviations are used in this manuscript:

AMR	Antimicrobial resistance
ARB	Antibiotic-resistant bacteria
ARG	Antibiotic resistance gene
BET	Brunauer–Emmett–Teller
BJH	Barrett–Joyner–Halenda
CoFe_2_O_4_	Cobalt ferrite
CW	Constructed wetland
CW-C	Biochar substrate
CW-CF	Biochar–zeolite composite substrate
CW-F	Zeolite substrate
CW-GCF	CoFe_2_O_4_-modified biochar–zeolite composite substrate
CW-LS	Gravel substrate
DNA	Deoxyribonucleic acid
EDTA	Ethylenediaminetetraacetic acid
HPLC	High-performance liquid chromatography
LC-MS/MS	Liquid chromatography–tandem mass spectrometry
LEfSe	Linear discriminant analysis effect size
MGE	Mobile genetic element
OTC	Oxytetracycline
OTU	Operational taxonomic unit
PCR	Polymerase chain reaction
qPCR	Quantitative real-time polymerase chain reaction
SEM	Scanning electron microscopy
SPE	Solid-phase extraction
SRM	Selected reaction monitoring
VFCW	Vertical-flow constructed wetland
XRD	X-ray diffraction
16S rRNA	16S ribosomal RNA
NCBI	National Center for Biotechnology Information
SRA	Sequence Read Archive
ICDD	International Centre for Diffraction Data
PDF-4+	Powder Diffraction File database
