# Supplementary material for: CoFe2O4-Modified Biochar Is Associated with Microbial Community and Antibiotic Resistance Gene Profiles in Vertical Flow Constructed Wetlands Exposed to Oxytetracycline
Source: Microorganisms. 2026 Sep 3;14(9):1950. doi: 10.3390/microorganisms14091950 (PMC13609335; doi:10.3390/microorganisms14091950)
Supplement: Supplementary file 1 [file microorganisms-14-01950-s001.zip › microorganisms-4518333-supplementary.pdf]

Table S1. Composition of the synthetic domestic wastewater used throughout the vertical flow constructed wetland experiment[1]

Category	Substance	Chemical Formula	Concentration (mg L^{-1})
Carbon source	Glucose	$C_6H_{12}O_6$	128.0
Carbon and organic nitrogen source	Peptone	n/a	118.0
Carbon source	Sodium Acetate	CH_3COONa	30.0
Inorganic nitrogen source	Ammonium Chloride	NH_4Cl	19.0
Phosphorus source	Potassium Dihydrogen Phosphate	KH_2PO_4	17.5
Mineral salt	Magnesium chloride	$MgCl_2$	6.0
Mineral salt	Calcium chloride	$CaCl_2$	2.0
Trace element	Copper sulfate pentahydrate	$CuSO_4{\cdot}5H_2O$	0.025
Trace element	Boric acid	H_3BO_3	6.2
Trace element	Manganese sulfate monohydrate	$MnSO_4{\cdot}H_2O$	6.37
Trace element	Zinc sulfate heptahydrate	$ZnSO_4{\cdot}7H_2O$	10.75
Trace element	Cobalt chloride	$CoCl_2$	0.025
Trace element	Sodium molybdate dihydrate	$Na_2MoO_4\ 2H_2O$	0.25

[1] Oxytetracycline was added separately to the synthetic wastewater at concentrations of 100, 200, or 500 μg L^{-1} according to the experimental phase described in Section 2.4.

Table S2 Mobile phase gradient elution program

Time (min)	A (%)	B (%)	flow rate mL / min
0.00	95	5	0.3
2.00	85	15	0.3
4.00	75	15	0.3
6.00	75	25	0.3
7.00	5	95	0.3
7.10	95	5	0.3
9.00	95	5	0.3

Table S3 qPCR Primer sequences, resistance mechanisms, and annealing temperatures [25]

Target gene	Primer sequence（5′→3′）	Annealing temperature (°C)	Drug resistance mechanism
16S rRNA	338F：GAGTTTGATCCTGGCTCAG<br>518R：ATTACCGCGGCTGCTGG	60	NA
aac(6′)-Ib	GTTTGAGAGGCAAGGTACCGTAA<br>GAATGCCTGGCGTGTTTGA	55	deactivate
*aad*A	CGAGATTCTCCGCGCTGTA<br>GCTGCCATTCTCCAAATTGC	54	deactivate
*tet*X	AGCCTTACCAATGGGTGTAAA<br>TTCTTACCTTGGACATCCCG	60	deactivate
*tet*A	GCTGTTTGTTCTGCCGGAAA<br>GGTTAAGTTCCTTGAACGCAAACT	54	efflux
*erm*B	GAGATCGGRCCAGGAAGC<br>GTGTGCACCATCGCCTGA	61	protection
*sul*1	CCGTTGGCCTTCCTGTAAAG<br>TTGCCGATCGCGTGAAGT	60	protection
*qnr*A	TTACTGCATCCATACGCTGCT<br>TATTTCTCACGCCAGGATTTG	54	unknown
bla_{CTX-M}	GGAGGCGTGACGGCTTTT<br>TTCAGTGCGATCCAGACGAA	56	deactivate
*int*I1	CCTCCCGCACGATGATC<br>TCCACGCATCGTCAGGC	60	Class1integron
*int*I2	GTTATTTTATTGCTGGGATTAGGC<br>TTTTACGCTGCTGTATGGTGC	55	Class2integron
*ISCR*1	CGCCCACTCAAACAAACG<br>GAGGCTTTGGTGTAACCG	52	transposase
